# Draft Genome Sequence of Bacillus lehensis M136, Isolated from the Manleluag Hyperalkaline Spring in Pangasinan, Philippines

**DOI:** 10.1128/MRA.00029-19

**Published:** 2019-03-28

**Authors:** Aprill P. Manalang, Andrew D. Montecillo, Lorren Myriccar G. Berdos, Rose Ann G. Franco, Eula Francia M. Bosito, Noel G. Sabino, Maria Genaleen Q. Diaz, Nacita B. Lantican

**Affiliations:** a Institute of Biological Sciences, University of the Philippines Los Baños College, Laguna, Philippines; University of Maryland School of Medicine

## Abstract

Here, we report the draft whole-genome sequence of Bacillus lehensis M136, isolated from a hyperalkaline spring located in Pangasinan, Philippines. From 24 scaffolds, the total genome assembly length is 3,985,437 bp. Industrially important genes like cyclodextrin glycosyltransferase (CGTase) and proteases were detected in this draft genome.

## Abstract (precis)

Here, we report the draft whole-genome sequence of Bacillus lehensis M136, isolated from a hyperalkaline spring located in Pangasinan, Philippines. From 24 scaffolds, the total genome assembly length is 3,985,437 bp.

## ANNOUNCEMENT

Bacillus lehensis, an alkalitolerant bacterium, was initially reported as a novel species by Ghosh et al. (1) following its isolation from soil samples in Leh, India. Its ability to produce enzymes such as cyclodextrin glycosyltransferase (CGTase) (2), maltogenic amylase (3), and protease (1) is of interest due to its many industrial applications. In this paper, we report the whole-genome sequence of *B. lehensis* M136 (C1-1), isolated from Manleluag Hyperalkaline Spring in Pangasinan, Philippines. The recorded pH range of the sampling site was pH 10 to 11.3, and the temperature ranged from 33 to 36°C. This strain is characterized by a “rotten egg” odor and blue flame ignition from gas bubble emissions, indicative of the high methane and sulfur content. The presence of serpentinizing seeps was also observed at the site. A water sample from the spring was collected and enriched using Horikoshi I medium, which generally supports growth of alkaliphilic and alkalitolerant bacteria. The putative *B. lehensis* M136 isolate, grown in Horikoshi I broth for 48 hours at 37°C, was sent for processing at the Philippine Genome Center DNA Sequencing Core Facility (Quezon City, Philippines). Genomic DNA was extracted using the Purelink genomic DNA extraction kit (Life Technologies Corporation, Carlsbad, CA, USA). Using 56.5 ng of DNA, a paired-end library was prepared using the Nextera XT library preparation kit and was sequenced using the Illumina MiSeq v3 2 × 300-cycle kit (Illumina, Inc., San Diego, CA, USA).

A total of 733,980 raw paired-end reads were generated. Quality trimming, carried out with the Trimmomatic tool (4) (using the following parameters: leading, 3; trailing, 3; slidingwindow, 4:15; minlen, 36; and headcrop, 10), produced 656,232 paired-end reads with a length of 26 to 291 bp. *De novo* assembly was performed using SPAdes version 3.11.1 (5) following default parameters and the “–careful” option to reduce the number of mismatches and short indels. Initial assembly produced 131 contigs, with the largest contig length of 1,848,692 bp and an *N*_50_ of 846,478 bp. To scaffold and gap fill the assembly, assembly improvement software (6) was used with default parameters. The assembly quality was analyzed using QUAST 4.6 (7), after which contigs with less than 500 bp and low coverage (<2.0×) were removed. The taxonomic classification of the isolate was established through Microbial Genome Atlas (MiGA) (8) analysis using the NCBI RefSeq and Prokaryotic databases. The contigs were then reordered using Mauve (9) Contig Mover with *B. lehensis* G1 NZ (GenBank accession number CP003923) as the reference. The draft genome sequence was annotated using the National Center for Biotechnology Information (NCBI) Prokaryotic Genome Annotation Pipeline (PGAP) (10) and the Rapid Annotations using Subsystems Technology (RAST) version 2 RASTtk pipeline (11, 12).

The B. lehensis M136 genome was found to contain 24 scaffolds and 29 contigs, including 5 gaps, for a total of 3,985,437 bp and a 39.8% G+C content (*N*_50_, 846,478 bp; coverage, 216×). Based on MiGA, the genome was found to be most similar to the genome of Bacillus lehensis G1 NZ (CP003923) (99.12% average nucleotide identity [ANI]), which was used in the contig reordering.

The NCBI PGAP predicted 3,923 protein-coding sequences, 14 rRNAs, 72 tRNAs, and 5 noncoding RNAs (ncRNAs). RASTtk annotation revealed the presence of genes for industrially important enzymes like CGTase (EC 2.4.1.19) and several proteases in this draft genome of *B. lehensis* M136.

### Data availability.

The genome sequence of Bacillus lehensis M136 has been deposited at DDBJ/ENA/GenBank under the accession number RQRY00000000 and SRA accession number PRJNA506874. The version described in this paper is version RQRY01000000.
